# Supplementary material for: Botulinum toxin in the treatment of partially accommodative esotropia with high AC/A ratio
Source: PLoS One. 2020 Feb 28;15(2):e0229267. doi: 10.1371/journal.pone.0229267 (PMC7048305; doi:10.1371/journal.pone.0229267)
Supplement: Attachment — Submitted filename: Review 9 Dec 19.docx [file pone.0229267.s002.docx]

Re: Manuscript PONE-D-19-32474 "Botulinum toxin in the treatment of high AC/A ratio accommodative esotropia".

I recommend that the article be accepted with Minor(?) revision. English is not the authors mother tongue, and the language needs to be corrected in many areas to diminish ambiguity. (While nevertheless appreciating that it is not easy to write in a language different to one’s home language)

The study is retrospective and comparative, and includes patients with accommodative esotropia(ET)[Because of the residual deviation despite wearing the full cycloplegic correction,this should be changed to partially accommodative ET{PAET} with a high accommodative convergence/accommodation ratio (AC/A).]The patients were divided into two groups. Group one patients were treated by botulinum toxin injection to both medial rectus muscles. Group two patients were treated by bilateral medial rectus muscle recessions, augmented with posterior fixation. The motor and sensory outcomes of patients in each group were compared.

While the manuscript is technically sound in most respects, I am concerned about the following: Patients received glasses based on the cycloplegic refraction at the initial visit, which were then worn for two months. The ET angle was measured at the next visit (the baseline visit). At that visit patients who had a residual distance ET of at least 10 prism dioptres (PD), and an ET at near that was larger than the distance ET by 10 PD or more were considered eligible for inclusion in Group 1 or 2(Lines 108-112).

It is considered by many that if the glasses do not reduce the distance deviation to less than 10 PD, another cycloplegic refraction, which usually discloses additional hyperopic refractive error, should be performed. If this is found, the spectacle power should be increased, and the glasses worn for at least another 4 weeks. If the new spectacles reduce the ET angle the refraction should again be repeated. This process may reduce or eliminate the distance deviation ,and reduce the distance/ near disparity.

From the manuscript it appears as if this was not done.(Lines 51and 52-“Cycloplegic refraction was carried out at the initial visit, at 6 months and a year after BTX injection or surgery”) Could the authors please comment?

Line 82 -88

Consider changing to “ …….with additional procedures, several of which are available………….deviation at near. We had used the later procedure for several years,………

for this condition.”

Line 85

Throughout the whole manuscript, change “medial recti and medial recti muscles” to “medial rectus muscles”, and change “bimedial recession” to “bilateral medial rectus muscle recessions”.

Line90.

“… parents did not agree to surgery. The results ………………..were considered to be satisfactory.”

Line104 .

Suggest change to “After a detailed explanation of the nature of the study, informed consent was obtained for BTX injection or surgery, and for collection of the relevant data……………”

Line 125.

Children with “………….myopia greater than -0.50D were excluded.” Were any myopic children include in the study? ( Line 174 “or myopic whose visual acuity improved with correction” )[Table 1 appears to indicate all patients were greater than +2.25 D hyperopic])

Line 133 .

Cycloplegic retinoscopy ………were also part of the study.(but only “at the initial visit, at 6 months and a year after BTX injection or surgery”) ?

Line 148.

Suggest change to: “…of the muscle pulley. Although this was identified during surgery,we nevertheless chose to use a classical scleral fixation technique to secure the muscle, with a non-absorbable 5-0 Dacron suture, and not a modified pulley fixation technique, as described by Clarke et al^5^”(published in 2004 ,and not later).

Line 158 -160

This is repetitive of lines 134-136

Lines 187 -192

Suggest change to “Six months after Botox injection or surgery, the deviation (with distance glasses) at distance and near, as well as stereoacuity were similar in the two groups. However, the deviations in the surgery group were significantly larger and stereoacuity in the BTX group significantly better at I year.”

Lines 191-192 states “stereoacuity was significantly better in the surgery group at I year”, while lines 238-239 and 350 (the legend to figure 2) state it was “better in the BTX group”. Please correct

The stereoacuity levels may have been related the duration of alignment before the constant ET developed(not studied)

Line 202-208.

“The percentage of success ….was similar in both groups……. at 6 months, but smaller in the surgery group at I year……...”

Table 1 contains the preintervention median, minimum and maximum refractions. Line 52 indicates that cycloplegic refractions were carried at the initial visit and 6 and 12 months after BTX or surgery. The manuscript does not report if any of the patients required a stronger hyperopic correction between the 6 and 12 month post- treatment visits, or in those requiring a new procedure (line 214). Any under correction could have influenced the deviations, stereoacuity, distance near disparity and the need for bifocals. As the authors point out (lines 193-196), whether statistically significant or not, most differences were of little clinical relevance

Line 255.

Replace with “We had no overcorrections in either study group. In the past, while performing larger recessions of x mm (authors to add in size of recessions), combined with posterior fixation we had overcorrections. These frequently increased with time and required reoperation. In the present study we thus adhered strictly to the AAO guidelines ^6^ ”

Line 279-284.

Could the authors comment on whether they could either slightly increase the amount of medial rectus muscle recession, or alter placement of the PFS, in order to improve the result of surgery with PFS without a greater risk of overcorrection

Line 280

By “more conservative(i.e.,less posterior)scleral……..” Do they mean placing the PFS more anteriorly, which should have less effect on the near deviation, but not on the distance deviation .If so how would it reduce the overcorrection rate?

955 Words
